# Open MRI validation of a hip model driven with subject-specific motion capture data in predicting anterior femoroacetabular clearance

**DOI:** 10.1186/s12891-021-04820-6

**Published:** 2021-11-23

**Authors:** Maryam Mohtajeb, Jolanda Cibere, Angelo Graffos, Michelle Mony, Honglin Zhang, Michael A. Hunt, David R. Wilson

**Affiliations:** 1 School of Biomedical Engineering, University of British Columbia, Vancouver, BC Canada; 2 Center for Hip Health and Mobility, University of British Columbia, Vancouver, BC Canada; 3 Department of Medicine, University of British Columbia, Vancouver, BC Canada; 4 Arthritis Research Centre of Canada, Richmond, BC Canada; 5 Motion Analysis and Biofeedback Laboratory, University of British Columbia, Vancouver, BC Canada; 6 Department of Physical Therapy, University of British Columbia, Vancouver, BC Canada; 7 Department of Orthopaedics, University of British Columbia, 7/F, 2635 Laurel Street, Vancouver, BC V5Z1M9 Canada

**Keywords:** Biomechanics, Hip, Femoroacetabular impingement, Mechanics, Model, Motion analysis

## Abstract

**Background:**

Cam and/or pincer morphologies (CPM) are potential precursors to hip osteoarthritis (OA) and important contributors to non-arthritic hip pain. However, only some CPM hips develop OA and/or pain, and it is not clear why. Anterior impingement between the femoral head/neck contour and acetabular rim during motion is a proposed pathomechanism. Understanding how activity and deformity combine to produce impingement may shed light on the causes of hip degeneration/pain. The objective of this study was to determine the accuracy of a subject-specific hip model driven by subject-specific motion data in predicting anterior impingement.

**Methods:**

We recruited 22 participants with CPM (both with and without pain) and 11 controls. We collected subject-specific 3D kinematics during squatting and sitting flexion, adduction, and internal rotation (FADIR) (an active and a passive maneuver, respectively, proposed to provoke impingement). We then developed 3D subject-specific hip models from supine 3T hip MRI scans that predicted the beta angle (a measure of anterior femoroacetabular clearance) for each frame of acquired kinematics. To assess the accuracy of these predictions, we measured the beta angle directly in the final position of squatting and sitting FADIR using open MRI scans. We selected the frame of motion data matching the static imaged posture using the least-squares error in hip angles. Model accuracy for each subject was calculated as the absolute error between the open MRI measure of beta and the model prediction of beta at the matched time frame. To make the final model accuracy independent of goodness of match between open MRI position and motion data, a threshold was set for least-squares error in hip angles, and only participants that were below this threshold were considered in the final model accuracy calculation, yielding results from 10 participants for squatting and 7 participants for sitting FADIR.

**Results:**

For squatting and sitting FADIR, we found an accuracy of 1.1°(0.8°) and 1.3°(mean (SD), and root mean squared error, respectively) and 0.5°(0.3°) and 0.6°, respectively.

**Conclusion:**

This subject-specific hip model predicts anterior femoroacetabular clearance with an accuracy of about 1°, making it useful to predict anterior impingement during activities measured with motion analysis.

## Introduction

Cam and pincer morphologies (CPM), which describe, respectively, an aspherical femoral head/head-neck junction and acetabular over-coverage, are potential precursors to hip osteoarthritis [1–8] and important contributors to non-arthritic hip pain [9–12]. However, only a subset of hips with CPM develops symptoms, which are primarily position-related pain in the hip and/or groin [13]. It is not clear which factors besides CPM are associated with symptoms and/or osteoarthritis. Impingement between the femoral head-neck junction and acetabular rim in extreme hip rotations is a proposed pathomechanism in femoroacetabular impingement (FAI) [1]. Impingement is sometimes assessed intraoperatively, but identifying impinging regions, especially arthroscopically, is technically challenging, and findings can be different from actual impingement occurring during in-vivo hip articulation in daily activities [14].

Biomechanical studies have examined hip rotational kinematics and kinetics during level walking [15–17] and other functional activities [18–21] in participants with CPM. A systematic review and meta-analysis [22] of these studies concluded that CPM hips with symptoms have lower peak hip extension angle, peak hip internal rotation angle, and external rotation hip torque during walking compared to control hips. For other activities, the review concluded that CPM hips with symptoms squatted to a lower depth compared to control hips with no differences in peak hip angles in all three planes at maximum squat depth. Evidence regarding the pelvic range of motion and other studied tasks like stair ascent was insufficient for any conclusion. It is not clear whether these observed alterations in hip kinematics and kinetics are directly caused by impingement or whether they are related to other mechanisms because direct measurement of femoroacetabular relationships cannot be made using these methods.

Impingement has been studied using finite element modeling of the hip to calculate mechanical stresses within the joint. Maximum shear stresses (MSS) in the acetabular cartilage and underlying bone in standing and maximum squat positions were calculated in a finite element study using hip models from two participants with severe cam deformity and two matched controls [23]. Inputs for each finite element model, including hip angles and net hip joint forces, were acquired from each participant’s motion capture data during a squat test. While similar cartilage MSS peaks were found in cam hips for standing (3.9 MPa) and squatting (3.6 MPa), MSS peaks of the underlying bone in cam hips were up to 4.7 times higher for squatting compared to standing. Further work is required to develop a robust finite element model of the hip that can predict impingement [24]. While the advantage of finite element studies is that they predict mechanical stresses, these models require many inputs (e.g., material properties, geometry, movement) that make validation and studies of large numbers of participants difficult.

Other studies have combined imaging and joint movement data to predict joint angles where contact is reached and/or contact locations on the femur and acetabulum [25–28]. One study assessed bony impingement points for simulated movements applied to 3D hip models constructed from CT data from 10 symptomatic cam hips, 10 asymptomatic cam hips, and 10 control hips [25]. In symptomatic cam hips, bony impingement occurred at significantly reduced internal rotation compared to asymptomatic cam hips and control hips. Unexpectedly, a significant number of identified impingement points occurred at the anteromedial part of the femoral neck rather than at the cam lesion. Impingement at the anterior part of the femoral neck was also observed for healthy control hips [25]. Another study that assessed bony impingement for applied simulated movements in models of 8 symptomatic CPM hips found that impingement regions could not be predicted using measurements of hip bony architecture: zones of initial contact did not correspond to the regions with minimum femoral head-neck offset in every case [27]. One limitation of this modeling approach is that it didn’t consider subject-specific kinematics data required to describe the subject-specific differences due to the unique neuromuscular, capsuloligamentous, and bony contributions to the motion of each patient. This limitation was addressed in a study using subject-specific motion data collected using a magnetic-based kinematics system and applied to 3D models of 13 cam hips [28], which found abutment of different regions of cam morphology against the anterosuperior quadrant of the acetabulum during various maneuvers. A limitation of these modeling studies is that, because few direct measurements of three-dimensional hip anatomy have been made at high hip rotation angles, these models have not been validated fully. None of these studies used subject-specific kinematics data collected using motion capture systems that are the current standard (state of the art) for studying human kinematics.

Our understanding of the role of activity in hip impingement might be improved by using subject-specific motion data to drive models with subject-specific hip anatomy from imaging. The motion data could include a large range of dynamic daily activities. Validating such models require three-dimensional visualization of the hip in a range of postures.

*Research Question*: What is the accuracy of a subject-specific hip model driven by subject-specific motion data in predicting anterior femoroacetabular clearance (impingement) during squatting and sitting flexion, adduction, and internal rotation (FADIR) maneuvers?

## Method

To assess the accuracy of subject-specific hip model predictions of anterior femoroacetabular clearance for squatting and sitting FADIR, we compared model predictions to direct measurements of anterior femoroacetabular clearance made using open MRI scans of the hips in the same postures. These two maneuvers require extreme hip angles, which are proposed to provoke impingement. Squatting represents an active impingement provoking maneuver while sitting FADIR represents a passive impingement provoking posture.

### Participants

We recruited 33 participants aged 28–56 years old, including 9 with cam and/or pincer morphology and with pain (CPM+), 13 with cam and/or pincer morphology and without pain (CPM-), and 11 controls. All participants were recruited from the original Investigation of Mobility, Physical Activity, and Knowledge Translation in Hip Pain (IMPAKT-HIP) cohort.

The IMPAKT-HIP cohort is a population-based sample of 500 Caucasian people recruited through random-digit dialing of households in greater Vancouver [29, 30]. In all participants, morphology of the femoral head-neck contour and acetabular coverage were assessed using supine Dunn view radiographs of the hip and standardized weight-bearing anteroposterior radiographs of the pelvis [31]. Hips were identified as having cam morphology if the alpha angle [32] was greater than 55° on the Dunn view radiograph [33]. Hips were identified as having pincer morphology if they had a lateral center edge (LCE) angle [34, 35] greater than 40° and/or a positive cross-over sign on the anteroposterior radiograph [31]. Hips identified as having both cam and pincer morphologies were classified as mixed. The presence of hip pain was defined as participant-reported pain in the groin and/or upper thigh lasting for 6 weeks or more and/or for 3 or more episodes during the past 12 months. This definition was designed to exclude pain due to soft tissue injuries/deficiencies and identify pain originating only from the hip. The study hip was defined as the hip with radiographic CPM. If CPM was present in both hips, then the hip with more severe pain was defined as the study hip. If equal or no hip pain was reported, the study hip was randomly selected. Groups were defined as follows: CPM+ hips were positive for the presence of pain and had at least one radiographic CPM; CPM- hips were negative for the presence of pain and positive for at least one radiographic CPM; Controls were negative for the presence of pain and had no radiographic CPM. Recruitment and screening for the original IMPAKT-HIP study spanned 1.5 years.

For our current study, which occurred a mean (SD) of 5.7(0.5) years after the original IMPAKT contact, phone screening was completed to recruit participants. The same definitions as the original IMPAKT study were used for CPM, hip pain, and the study hip. New exclusion criteria were considered for the current study, including previous lower limb surgeries, injuries, or any neurological conditions that affected everyday recreational or sporting activities over the past 12 months, a history of any inflammatory or autoimmune diseases, avascular necrosis of the hip, planned or previous lower limb joint replacement, or physician-diagnosed lower limb joint osteoarthritis. The Clinical Research Ethics Board of the University of British Columbia approved the study, written informed consent was obtained from all participants, and the study was conducted in accordance with the Declaration of Helsinki.

### Motion analysis

We collected 3D kinematics data of each participant during squatting and sitting FADIR maneuvers at 120 Hz using a fourteen-camera motion capture system (Motion Analysis Corporation, Santa Rosa, CA). Fifty passive retroreflective markers applied to various bony landmarks were used for motion tracking. Bilateral bony landmarks included the acromioclavicular joint, posterior superior iliac spine (PSIS), iliac crest, anterior superior iliac spine (ASIS), greater trochanter, anterior thigh, lateral and medial femoral epicondyle, anterior shank, lateral and medial malleoli, posterior calcaneus, medial aspect of the head of the 1st metatarsal bone, dorsal aspect of the head of the 2nd metatarsal bone, and the lateral aspect of the head of the 5th metatarsal bone. Other bony landmarks included vertebra C_7_, vertebra T_10_, right scapula, and the sternal notch. Finally, cluster plates of 4 markers were applied bilaterally on the shanks and thighs to track the movements of their respective segments during the squatting and sitting FADIR maneuvers. A static standing trial with the same foot distance and orientation as the open MRI standing posture was then conducted. After the static standing trial was completed, the medial epicondyle and malleoli markers were removed for the squatting and sitting FADIR trials.

#### Squatting

**Fig. 1:**
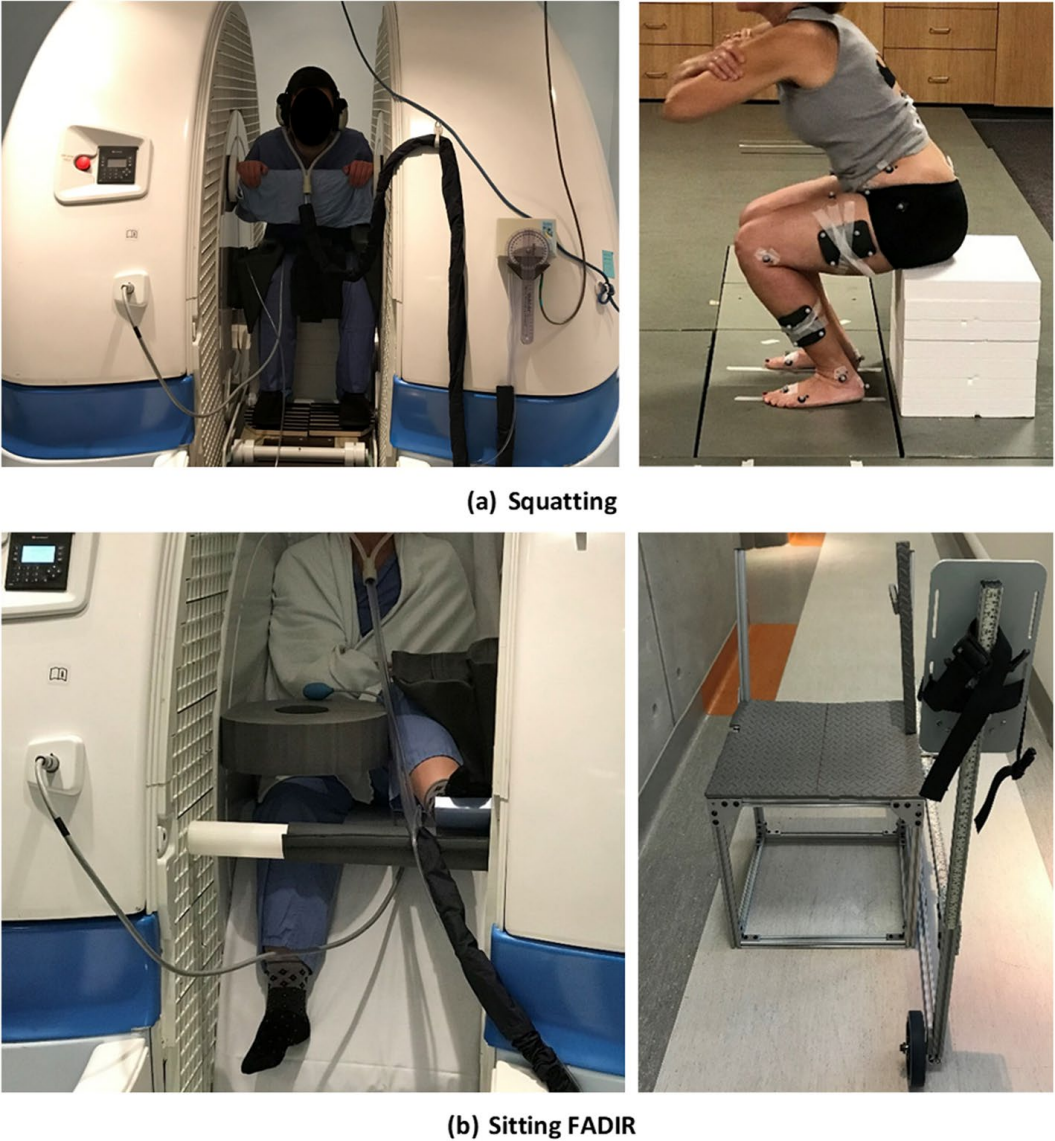
Positioning at the MROpen scanner and motion capture lab for (**a**) squatting and (**b**) sitting FADIR

Participants were positioned with their feet oriented anteriorly and with the most lateral aspects of the toes 22 cm apart and their arms crossed across their chest. They squatted as deeply as they could without rotating or lifting any part of their feet (Fig. 1 (a)). Maximum squat depth was measured for each participant from the ground to the lowest part of the buttocks. Motion data for 5 trials were recorded for each participant. The toe distance restriction was applied because the final position of the squatting maneuver performed in the motion analysis lab needed to be replicated in the open MRI scanner during the scanning and was dictated by the open MRI scanner bore dimensions and the necessity of positioning the study hip at the scanner isocenter.

#### Sitting FADIR

For sitting FADIR, the participant’s study side hip was moved into the FADIR pose using a chair designed to match the bore and chair dimensions of our open MRI scanner (Fig. 1 (b)). The horizontal distance between the mid-point of the chair seat and the most lateral margin of the chair foot holder is equal to the horizontal distance between the mid-point of the open MRI chair and open MRI wall. Participants were positioned with their study hip in the middle of the chair while their foot was secured in the chair foot holder.

The chair design allowed each participant’s hip and foot to be positioned and constrained as it was within the open MRI scanner (Fig. 1 (b)) for the same posture. The study hip was flexed and then adducted and internally rotated to the maximum limit the participant could tolerate for the duration of scanning (about 30 min) using foot displacement in the horizontal and vertical direction and then moving the knee toward the body mid-line. Motion data for 5 trials were recorded for each participant.

### 3D hip models

To develop 3D subject-specific hip models, we scanned each participant’s study hip in supine using a sequence optimized for hip cortical bone visualization in a 3T MRI scanner (Achieva, Philips, Eindhoven, Netherlands) (Table 1). 3T scanning of the hips was performed a mean (SD) of 4.2 (0.54) years prior to the current study and spanned 1.08 years. Subject-specific 3D models (point clouds) of the femur and acetabulum were developed by segmenting these bones manually from 3T MRI scans using Analyze software (AnalyzeDirect, Inc., Overland Park, KS, US).

**Table 1:** Sequence details of 3T MRI hip scans used for developing 3D hip models and MROpen hip/pelvis/knee scans used for defining a hip joint coordinate system in supine/standing and calculating hip angles in squatting/sitting FADIR in the MROpen

	Sequence	Matrix	FOV (field of view)	Slice Thickness
3T MRI Hip scans	TSE^1^ TE^2^/TR^3^ = 10 ms/776.7 ms	512 × 512	20 cm × 20 cm	2 mm
MROpen Hip Alpha Plane scans	GFE^4^ short TE, TE/TR = 8 ms/443 ms	256 × 256	25 cm × 25 cm	2.5 mm (0.5 mm gap)
MROpen Hip Sagittal Scans	GFE, short TE sequence, TE/TR = 8 ms/627 ms	256 × 256	25 cm × 25 cm	2.5 mm (0.5 mm gap)
MROpen Pelvis Axial Scans	GFE, TE/TR = 12 ms/370 ms	256 × 256	30 cm × 30 cm	2.5 mm (0.5 mm gap)
MROpen Knee Axial Scans	GFE, TE/TR = 8 ms/650 ms	256 × 256	20 cm × 20 cm	2.5 mm (0.5 mm gap)

^1^Turbo spin echo, ^2^. Echo Time, ^3^. Repetition Time, ^4^. Gradient Field Echo

### Open MR imaging

We scanned each participant’s study hip in supine, standing, squatting, and sitting FADIR poses with an upright open MRI scanner (MROpen, Paramed, Genoa, Italy) to a) measure impingement directly and b) measure hip angles. To measure impingement, we acquired scans in planes parallel to the femoral neck and perpendicular to the femoral neck and femoral shaft axes (alpha plane) (Table 1) in squatting and sitting FADIR. For hip angles, we acquired images in the sagittal plane (Table 1) for supine, squatting, and sitting FADIR. We also scanned each participant’s pelvis and study side knee in supine to define a hip joint coordinate system.

We applied the following protocols for the squatting and sitting FADIR postures in the MROpen.

#### Squatting

The MROpen bed was adjusted, and several foam pads were placed on the bed to replicate the squat depth measured in the motion analysis lab for each participant. Participants were positioned so that the top foam was touching the buttocks to prevent participant motion and associated movement artifact. Foot orientation and position from the motion analysis lab were replicated in the scanner using a pair of sandals attached to a wooden plate. Several foam pads were put around the participants’ knees to minimize movement artifact (Fig. 1 (a)).

#### Sitting FADIR

Participants were positioned in the scanner chair with the study hip at the center of the scanner. The hip was flexed, adducted, and internally rotated to the same posture used in the motion analysis study. The foot was secured against the MROpen scanner wall using a support bar. The knee was supported with several foam pads to minimize motion artifact (Fig. 1 (b)).

### Hip angle measurement from MROpen

To calculate hip joint angles from the MROpen images, we first defined hip joint coordinate systems according to the International Society of Biomechanics (ISB) recommendations [36] using the supine scans. To define the hip joint coordinate system, a Cartesian coordinate system was first defined for both the femur and pelvis. The pelvis coordinate system was defined using the right and left anterior superior iliac spines (ASIS) and right and left posterior superior iliac spines (PSIS). Orientations of pelvis coordinate system unit vectors were as follows: Z-axis: parallel to the line connecting the right and left ASIS pointing laterally; X-axis: parallel to a line lying in the plane defined by right and left ASIS and the midpoint of the right and left PSIS, perpendicular to the Z-axis, pointing anteriorly; Y-axis: cross product of X and Z pointing superiorly. The origin of the pelvis coordinate system was defined as the center of the best fit sphere to the acetabulum lunate surface. The femur coordinate system was defined using medial and lateral femoral epicondyles and the center of the best fit sphere to the femoral head surface. Orientations of the femur coordinate system unit vectors were as follows: y-axis: the line joining the midpoint between the medial and lateral femoral epicondyles and the center of the best fit sphere to the femoral head pointing superiorly; z-axis: the line lying in the plane defined by the center of the best fit sphere to the femoral head and the medial and lateral femoral epicondyles perpendicular to y, pointing laterally; x-axis: cross product of y and z pointing anteriorly. The orientations of the hip joint coordinate system unit vectors are as follows: **e**_**1**_: Z-axis of the pelvis coordinate system fixed to the pelvis (flexion/extension); **e**_**2**_: y-axis of the femur coordinate system fixed to the femur (internal/external rotation); **e**_**3**_: cross product of **e**_**1**_ and **e**_**2**_, which is the floating axis (abduction/adduction). The origin of the hip coordinate system was considered the same as the femur coordinate system origin. Bony landmarks, including right and left ASIS/ right and left PSIS, and medial/lateral femoral epicondyles were identified from the supine scans of the pelvis and knee, respectively. The femoral head surface and acetabular lunate surfaces were segmented from the supine sagittal scans of the hip.

To calculate hip joint angles from the MROpen images of the squatting and sitting FADIR poses, 3D models of the femur and acetabulum in the supine, squatting, and sitting FADIR postures were created by segmenting sagittal scans of the hip for these postures. 3D models in supine were registered to 3D models for the squatting and sitting FADIR postures using the finite iterative closest point (ICP) algorithm [37]. Hip joint angles in each posture were calculated using the Grood and Suntay convention [38] applied to the hip joint coordinate system.

### Model validation

**Fig. 2:**
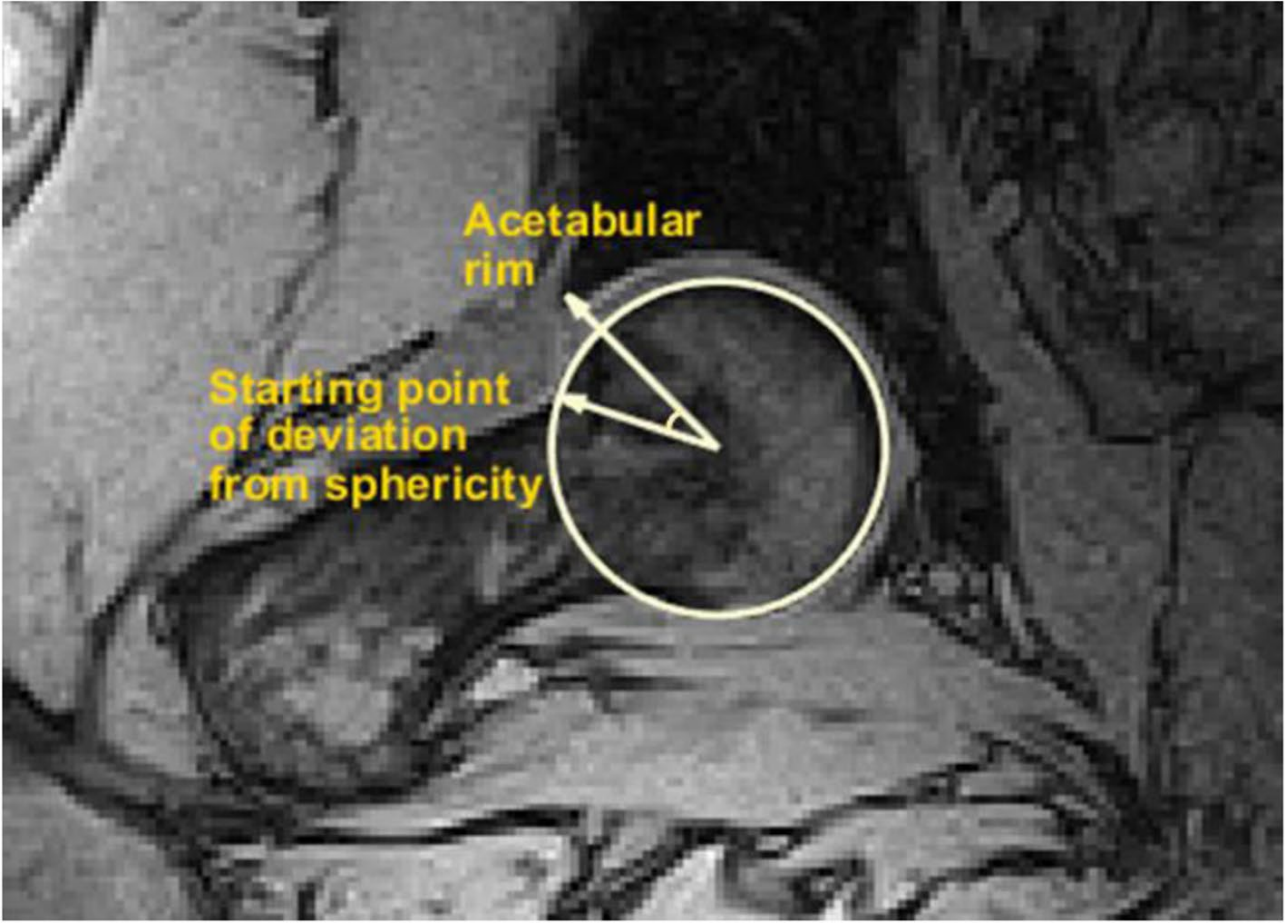
Illustration of beta angle in a control hip at the squatting posture

The primary outcome variable was the beta angle, which defines clearance between the femoral head/neck junction and the acetabular rim [39]. The beta angle is measured on the same imaging plane as the alpha angle and is defined as the angle between a line drawn from the femoral head center to the most lateral bony margin of the acetabular rim and a second line drawn from the center of the femoral head to the starting point of deviation from sphericity in the femoral head-neck contour (Fig. 2). There is a significant association between negative beta angle and elevated acetabular rim contact pressures [40].

We calculated hip joint angles from the motion analysis data using the same joint coordinate system. The position of the joint coordinate system (found in supine) in the standing posture was determined by using transformation matrices found by registering 3D hip models in supine to 3D hip models in standing created from sagittal hip scans in supine and standing, respectively. The locations of the motion analysis markers in the identical (standing) posture used in the MROpen scanner were determined. Given the location of the joint coordinate system and the motion analysis markers in this reference standing posture, the location of the joint coordinate system for any subsequent frame of motion analysis data could be calculated, and joint angles could be determined using the Grood and Suntay convention [38].

Hip position for the squatting and sitting FADIR postures imaged in the MROpen scanner was matched to the motion analysis data by choosing the frame of motion analysis data that yielded the minimum least-squares error between hip joint angles in the MROpen and motion analysis data.

For the squatting and sitting FADIR postures, we compared the model prediction of the beta angle to the direct measurement of the beta angle on the MROpen scan. We determined the hip joint angles for the squatting posture in the MROpen scanner and then identified the frame of squatting from the motion analysis data that best matched these joint angles (i.e., with least-squares error in hip joint angles). Femur and acetabulum 3D models segmented from the 3T scans were positioned to this identified time frame of motion sequence using calculated transformation matrices from the supine posture (corresponding to the 3T image acquisition) to the relevant frame of motion analysis data. The femur’s position was adjusted to match the femur and acetabulum center-to-center distance calculated from the 3T MRI scans. The orientation of the alpha plane was determined, and a series of planes parallel to the alpha plane and spaced 2.5 mm apart were passed through the positioned 3D model of the hip to replicate MROpen slices. The intersection of these planes with the hip 3D models was found as the points within 0.5 mm of the defined planes. The beta angle was calculated for each plane. The minimum beta angle (for all planes) was found and compared to the minimum beta angle measured from the MROpen scans.

### Statistical analysis

We calculated each subject-specific model accuracy as the absolute difference between the beta angle calculated from the hip model and the beta angle measured directly from the MROpen scans. Final model accuracy was described with the mean (SD) (and root mean squared) of subject-specific model accuracies.

We assessed the relationship between the least-squares error in hip angles and subject-specific model accuracy using the Pearson correlation coefficient (and intra-class correlation coefficient (ICC)) to better understand how model accuracy is related to how well the positions from the motion analysis data and the MROpen data are aligned.

To exclude data points where the accuracy would be affected by the difference in hip position between the MROpen posture and the motion analysis posture, we set a threshold for the least-squares error of hip angles, and only considered participants that were below this threshold in the final model accuracy calculation. This threshold was defined by including participants with the lowest least-squares error in hip angles until there was no statistically significant relationship between the least-squares error in hip angles and subject-specific model accuracy. For squatting, we had to include participants with less than 10% mean least-squares error in hip angles (10 participants) to reach no significant correlation (r = 0.47, *p* = 0.17) between the least-squares error of hip angles and subject-specific model accuracy (ICC = 0.041). For sitting FADIR, we had to include participants with less than 5% mean least-squares error in hip angles (7 participants) to reach no significant correlation (r = 0.63, *p* = 0.12) between the least-squares error of hip angles and subject-specific model accuracy (ICC = 0.0071) (3 participants who met the threshold criteria for the sitting FADIR were excluded from the final accuracy calculation because either they hadn’t completed the 3T MRI scanning or had very low-quality scans).

We assessed the relationship between model accuracy and participant body mass index (BMI) using the Pearson correlation (and ICC) to investigate how BMI affects the amount of skin-mounted marker movement relative to the hip bones and model accuracy.

## Results

**Fig. 3:**
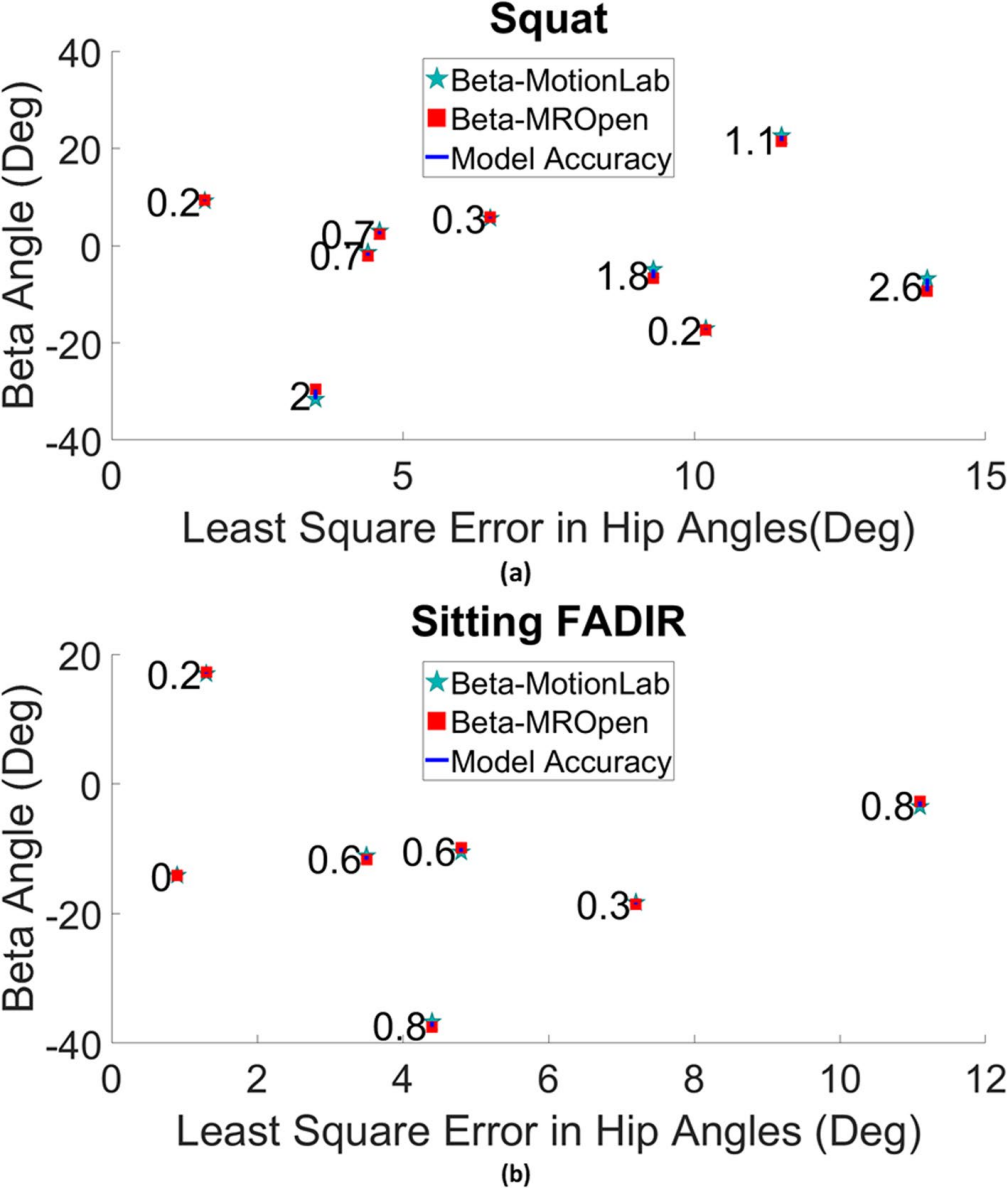
Beta measured from MROpen scans and beta calculated from subject-specific hip models (motion lab) and their absolute differences (label numbers) as the subject-specific model accuracy in predicting beta angle for (**a**) squatting and (**b**) sitting FADIR; in terms of the least-squares error in hip angles (for participants considered in the final accuracy calculation)

For squatting, the mean absolute error (SD) and root mean squared error (RMSE) between the model prediction of beta angle and the direct measure of beta angle from the MROpen were 1.1°(0.8°) and 1.3°, respectively (Table 2) (Fig. 3 (a)). We found no statistically significant correlation (r = − 0.25, *p* = 0.48) between the model accuracy and participant BMI for the squat (ICC = 0.047).

**Table 2:** Hip angles in the MROpen and motion analysis lab, least-squares error in hip angles, and corresponding beta angles for squatting (for the participants considered in the final accuracy calculation)

Participant #	Flex^a^, MoLab^b^ (°)	Flex, MROpen (°)	IR^c^, MoLab (°)	IR, MROpen (°)	Abd^d^, MoLab (°)	Abd, MROpen (°)	LSQE^e^ (°)	Beta MoLab (°)	Beta MROpen (°)	Absolute Difference in Beta (°)	BMI (kg/m^2^)
Control (1)	64.4	64.2	−2.3	−0.7	5.5	5.9	1.6	9.2	9.4	0.2	25.0
CPM+ (1)	69.5	68.0	14.4	17.5	5.7	6.0	3.5	−31.6	−29.6	2.0	27.5
CPM+ (2)	84.9	87.9	5.4	2.4	4.5	3.2	4.4	−1.4	−2.1	0.7	21.5
CPM-(1)	76.1	79.4	23.0	20.0	2.9	1.7	4.6	3.0	2.3	0.7	26.1
CPM-(2)	78.4	77.9	−8.2	−1.8	10.0	11.3	6.5	5.6	5.9	0.3	28.5
CPM-(3)	84.5	81.2	8.9	17.6	5.0	5.6	9.3	−4.9	−6.7	1.8	23.2
Control (2)	66.7	74.7	−0.7	−4.2	5.6	2.2	9.4	21.4	20.2	1.2	25.8
CPM+ (3)	63.7	64.7	−3.7	−7.6	−4.5	4.9	10.2	−17.1	−17.3	0.1	32.6
CPM-(4)	76.3	81.1	−3.7	6.2	5.7	8.9	11.5	22.6	21.5	1.1	32.8
CPM-(5)	95.4	101.6	16.2	4.2	7.4	6.5	13.6	−6.8	−9.4	2.6	25.0

^a^*Flex* Flexion, positive values represent flexion, and negative values represent extension; ^b^*MoLab* Motion lab; ^c^*IR* Internal rotation, positive values represent internal rotation, and negative values represent external rotation; ^d^*Abd* Abduction, positive values represent abduction, and negative values represent adduction; ^e^*LSQE* Least-squares error

For sitting FADIR, the mean absolute error (SD) and root mean squared error (RMSE) between the model prediction of beta angle and the direct measure of beta angle from the MROpen was 0.5° (0.3°) and 0.6°, respectively (Table 3) (Fig. 3 (b)). We found no statistically significant correlation (r = 0.51, *p* = 0.24) between the model accuracy and participant BMI for sitting FADIR (ICC = 0.004).

**Table 3:** Hip angles in the MROpen and motion analysis lab, least-squares error in hip angles, and corresponding beta angles for the sitting FADIR (for the participants considered in the final accuracy calculation)

Participant #	Flex^a^, MoLab^b^ (°)	Flex, MROpen (°)	IR^c^, MoLab (°)	IR, MROpen (°)	Abd^d^, MoLab (°)	Abd, MROpen (°)	LSQE^e^ (°)	Beta MoLab (°)	Beta MROpen (°)	Absolute Difference in Beta (°)	BMI (kg/m^2^)
CPM-(6)	89.4	88.9	7.3	7.9	8.6	8.0	0.9	−14.1	− 14.1	0.0	28.0
CPM-(7)	87.9	88.3	14.7	15.0	2.4	1.1	1.3	17.0	17.2	0.2	23.1
CPM-(1)	86.9	88.1	38.0	37.3	1.2	−2.0	3.5	− 11.1	− 11.7	0.6	26.1
CPM+ (1)	96.7	98.5	18.6	16.9	7.5	3.8	4.4	−36.7	− 37.5	0.8	27.5
CPM-(8)	90.8	93.8	19.2	17.2	6.6	3.4	4.8	− 10.5	−9.9	0.6	30.3
CPM+ (4)	85.5	84.9	12.4	16.5	0.9	6.9	7.2	− 18.3	− 18.6	0.3	19.3
CPM-(4)	88.7	81.6	16.3	23.6	−14.3	− 10.0	11.1	− 3.5	−2.7	0.8	32.8

^a^*Flex* Flexion, positive values represent flexion, and negative values represent extension; ^b^*MoLab* Motion lab; ^c^*IR* Internal rotation, positive values represent internal rotation, and negative values represent external rotation; ^d^*Abd* Abduction, positive values represent abduction, and negative values represent adduction; ^e^*LSQE* Least-squares error

## Discussion

In this study, we assessed the accuracy of a subject-specific hip model combined with motion analysis data in predicting the beta angle, a measure of hip clearance, by comparing model predictions to direct measurements made using open MRI. We found an accuracy of 1.1° for squatting and an accuracy of 0.5° for sitting FADIR.

This simple model allows subject-specific assessment of hip clearance using motion data combined with a supine and standing MRI scan of the hip and a supine scan of the pelvis and knee to identify required bony landmarks for defining the hip joint coordinate system. Our accuracy figures for beta angle prediction are useful for planning and interpreting studies using this model. Our correlation results for BMI show that his model is not affected by participant BMI within the range of BMIs that we tested. The model in this study can predict impingement without requiring assumptions such as simplified anatomy or averaged material properties for the bone or soft tissues.

The accuracy of our model is comparable to the accuracy of a combined dual fluoroscopy and model-based tracking approach used for studying kinematics of 3 CPM hips with symptoms and 6 control hips [41, 42]. This study reported a bias and precision of less than 1° using an ex-vivo cadaver study. Although highly accurate, the need for ionizing radiation and the somewhat limited imaging region associated with dual fluoroscopy make it impractical for assessing a large range of activities.

This model has the potential to provide valuable data that support and enhance what has been learned from finite element models of the hip [23, 43–45]. In these models, the geometry of hip structures, including the femur, acetabulum, cartilage, and labrum (if considered), were modeled using either subject-specific imaging data or more simplified geometry. Most of these studies assumed isotropic, linear elastic behavior for the cartilage and labrum; however, one study modeled the biphasic behavior of cartilage. Subject-specific kinematics and kinetics data were assumed in some but not all of these studies. Regardless of the simplifications and sophistication of the modeling approaches, all these studies showed that intrusion of the non-spherical femoral head-neck contour into the acetabulum (impingement) corresponds to elevated joint stresses. The increased stresses were larger at higher hip angles (i.e., reaching the terminal position of the studied maneuvers). The model from the current study can be used to predict when and to what extent this intrusion is happening during dynamic activities.

An advantage of our model over the dynamic CT models that are currently used clinically to predict impingement is that our model uses subject-specific kinematics data. Many dynamic CT models [25–27] rotate the femur relative to the acetabulum using generalized hip motions until femoroacetabular collision is detected, which is defined as the end of the hip range of motion, and the points of contact are considered as impingement regions. Biomechanical studies have shown that there are differences in hip and pelvic kinematics between hips with FAI syndrome and pain-free control hips, which demonstrates the importance of using subject-specific hip kinematics to predict impingement. One dynamic CT model [28] used subject-specific kinematics, but only from passive maneuvers of the hip. Further, we are not aware of direct validation of impingement predicted by these CT simulations against actual measures of impingement in*-*vivo.

This model could be implemented in clinical practice, but two essential steps would likely be required to make the procedure less time-consuming. First, we would modify the procedure to estimate the hip coordinate system from the external motion markers, which would enable us to run the model without the standing scan of the hip. This would require quantifying the effect of defining the hip coordinate system using the external motion markers rather than actual anatomical landmarks on the model accuracy. Second, we would automate the segmentation of hip bones from the MRI scans, which would be much faster than the manual segmentation used in this study. This would require quantifying the effect of automatic segmentation on the accuracy of the model.

One strength of the study is that we assessed model accuracy in-vivo using the population that this model was developed to study rather than in cadavers or control hips. Hip kinematics are influenced by hip morphology and pain status, and our study included CPM+, CPM-, and normal hips. Unfortunately, we did not have sufficient numbers of participants in each group to compare our accuracy findings between these groups. A second strength is that we assessed model accuracy during both passive (sitting FADIR) and active (squatting) maneuvers. Soft tissue behavior might be different during an active maneuver than a passive maneuver, which might affect hip kinematics and, therefore, model predictions. A final strength is that hip angles during the motion analysis maneuvers were calculated using the hip joint coordinate system built through the actual bony landmarks identified from the MROpen scans. This method is in contrast with the more widely used approach of considering retroreflective motion capture markers as actual bony landmarks and estimating the femoral head center using these external markers, which can be inaccurate because of the soft tissue layer between the external retroreflective markers and actual bony landmarks as well as errors in locating the bony landmarks.

One limitation of our study is that we validated this model for only two postures. However, these postures were chosen because they are likely to place the hip in an impinging position and therefore represent important positions for a model that assesses impingement. A second limitation is that the 3T scans of the hips were acquired a mean (SD) of 4.2 (0.54) years prior to our current study. This limitation is unlikely to affect our findings because the 3T scans were only used to describe the bony anatomy, and it has been shown that femoral head-neck anatomy in cam hips and acetabular coverage in dysplastic hips does not change over 5 years [46] and 20 years [47]. Another limitation of our study is that the 3T scans had a relatively large slice thickness (2 mm). An isotropic high-resolution scan of the hip could make the 3D representation of the hip joint closer to the actual morphology. A further limitation is that we did not assess the accuracy of this model for the more widely used case where the hip joint coordinate system is defined using external retroreflective motion capture markers instead of bony landmarks acquired from MRI. Finally, we assessed accuracy in only 10 participants for the squat and 7 participants for the sitting FADIR due to the challenges of closely matching MROpen to motion analysis postures, which, though somewhat limited, is higher than many other validation studies.

## Conclusion

We conclude that this subject-specific hip model driven by subject-specific motion data predicts beta angle (anterior femoroacetabular clearance) with an accuracy of about 1°, which makes it useful for predicting anterior impingement during activities measured with motion analysis.

## Data Availability

The datasets used and/or analysed during the current study are available from the corresponding author on reasonable request.

## Abbreviations

OA: Osteoarthritis
CPM: Cam and/or pincer morphologies
FADIR: Flexion, adduction, and internal rotation
RMSE: Root mean squared error
FAI: Femoroacetabular impingement
MSS: Maximum shear stresses
CPM+: Cam and/or pincer morphology with pain
CPM-: Cam and/or pincer morphology without pain
IMPAKT-HIP: Investigation of Mobility, Physical Activity, and Knowledge Translation in Hip Pain
MROpen: Open MRI scanner
PSIS: Posterior superior iliac spine
ASIS: Anterior superior iliac spine
ISB: International Society of Biomechanics
ICC: Intra-class correlation coefficient
